# Polyphasic evaluation of key cyanobacteria in biocrusts from the most arid region in Europe

**DOI:** 10.7717/peerj.6169

**Published:** 2019-01-03

**Authors:** Beatriz Roncero-Ramos, M. Ángeles Muñoz-Martín, Sonia Chamizo, Lara Fernández-Valbuena, Diego Mendoza, Elvira Perona, Yolanda Cantón, Pilar Mateo

**Affiliations:** 1 Departamento de Agronomía, Universidad de Almería, Almería, Spain; 2 Departamento de Biología, Universidad Autónoma de Madrid, Madrid, Spain; 3 Department of Agrifood Production and Environmental Sciences, University of Florence, Florence, Italy; 4 Centro de Investigación de Colecciones Científicas de la Universidad de Almería, Universidad de Almería, Almería, Spain

**Keywords:** Biological soil crust, Biocrusts, Soil cyanobacteria, Phylogenetic relationships, 16S rRNA gene

## Abstract

Cyanobacteria are key microbes in topsoil communities that have important roles in preventing soil erosion, carbon and nitrogen fixation, and influencing soil hydrology. However, little is known regarding the identity and distribution of the microbial components in the photosynthetic assemblages that form a cohesive biological soil crust (biocrust) in drylands of Europe. In this study, we investigated the cyanobacterial species colonizing biocrusts in three representative dryland ecosystems from the most arid region in Europe (SE Spain) that are characterized by different soil conditions. Isolated cyanobacterial cultures were identified by a polyphasic approach, including 16S rRNA gene sequencing, phylogenetic relationship determination, and morphological and ecological habitat assessments. Three well-differentiated groups were identified: heterocystous-cyanobacteria (*Nostoc commune*,* Nostoc calcicola*,* Tolypothrix distorta* and *Scytonema hyalinum*), which play an important role in N and C cycling in soil; nonheterocystous bundle-forming cyanobacteria (*Microcoleus steenstrupii*,* Trichocoleus desertorum*, and *Schizothrix* cf*. calcicola*); and narrow filamentous cyanobacteria (*Leptolyngbya frigida* and *Oculatella kazantipica*), all of which are essential genera for initial biocrust formation. The results of this study contribute to our understanding of cyanobacterial species composition in biocrusts from important and understudied European habitats, such as the Mediterranean Basin, a hotspot of biodiversity, where these species are keystone pioneer organisms.

## Introduction

Drylands represent approximately 40% of the Earth’s surface and are characterized by low vascular plant cover (Maier et al., 2014) surrounded by open spaces frequently colonized by biocrusts, which cover approximately 12.2% of the Earth’s surface (Rodríguez-Caballero et al., 2018). Biocrusts are complex topsoil microbial assemblages composed of cyanobacteria, microalgae, lichens, mosses, fungi, heterotrophic bacteria and archaea (Velasco Ayuso et al., 2017) living in close association with soil particles. Biocrusts increase biodiversity in many ecosystems (Belnap, Weber & Büdel, 2016), and several studies have proposed the use of biocrusts as model systems to study the link between biodiversity and ecosystem multifunctionality (Bowker et al., 2013; Bowker et al., 2014).

Cyanobacteria are biocrust community members that have been demonstrated to provide important soil functions. They are pioneers in the soil stabilization process (Garcia-Pichel & Wojciechowski, 2009) and increase microtopographic roughness (Rodríguez-Caballero et al., 2012), reducing erosion by water (Cantón et al., 2014) and wind (Zhang et al., 2008). In addition, cyanobacteria enhance water availability and soil stability (Chamizo et al., 2012b; Chamizo et al., 2016) via the filamentous morphology of some genera and the exopolysaccharides they excrete (Rossi & De Philippis, 2015). In some species, many individual filaments group together and are surrounded by a common sheath, forming bundles of filaments in a net-like structure that binds soil particles together, contributing to soil cohesion (Thomas & Dougill, 2007). In addition, apart from the capacity of cyanobacterial vegetative cells to fix atmospheric CO_2_, some specialized cells of heterocystous cyanobacteria, heterocysts (heterocytes), can fix atmospheric N_2_ (Belnap, 2003; Rodríguez-Caballero et al., 2018). Thus, cyanobacteria increase soil fertility and improve the retention of nutrients in the topsoil (Beraldi-Campesi et al., 2009). Cyanobacteria have many other adaptations that allow them to be the first colonizers of soil surfaces after a disturbance. They are able to tolerate high temperatures and UV radiation by producing UV-screening pigments, such as scytonemin (Garcia-Pichel & Castenholz, 1991; Potts, 1994). As the capacity of cyanobacterial species to colonize a particular soil surface depends to a great extent on environmental conditions, such as temperature (Garcia-Pichel et al., 2013; Muñoz-Martín et al., 2019), native species may have developed specific survival mechanisms for site-specific conditions (Weber et al., 2016, and references therein). Moreover, cyanobacteria migrate through the soil surface by lateral (Sorochkina, Velasco Ayuso & Garcia-Pichel, 2018) or vertical (Garcia-Pichel & Pringault, 2001) motility for dispersion and protection from high insolation. In addition, cyanobacterial metabolism is adapted to the typical hydration and desiccation cycles in deserts (Rajeev et al., 2013), becoming active when water is available, such as during dewfall events (Büdel et al., 2008). The association of cyanobacteria with other biocrust organisms, such as heterotrophic bacteria, through processes such as cross-feeding, has also been described (Baran et al., 2015). All these features make cyanobacteria pioneer organisms capable of successfully colonizing degraded soils and may be crucial in facilitating the succession of more developed organisms, such as mosses or lichens, as well as vascular plants in later stages (Weber et al., 2016, and references therein).

Studies of cyanobacterial diversity in biocrusts have shown different cyanobacterial community compositions depending on the geographical region. The bundle-forming genus *Microcoleus* has been observed to be dominant in arid and semiarid regions worldwide, including the most extreme environments, such as the hyperarid Atacama desert (Patzelt et al., 2014; Büdel et al., 2016; Baumann et al., 2018). A continental-scale compositional survey of biocrust cyanobacterial communities across arid and semiarid North America showed a latitudinal switch in the dominance of different species of *Microcoleus* (Garcia-Pichel et al., 2013). *Microcoleus vaginatus* dominates cool desert soils, whereas *Microcoleus steenstrupii* dominates hot arid to semiarid deserts. However, an analysis of the cyanobacterial communities of biocrusts across a latitudinal gradient of Western Europe showed that *Microcoleus* species were not dominant (Williams et al., 2016), and the filamentous cyanobacteria belonging to the family Leptolyngbyaceae were the most abundant in Arctic soil biocrusts (Pushkareva et al., 2015). In most cases, biocrusts are also dominated by filamentous cyanobacteria belonging to genera *Trichocoleus*, *Schizothrix*, *Leptolyngbya*, *Phormidium*, and other Phormidiaceae (e.g.,  Johansen, 1993; Garcia-Pichel et al., 2013; Dojani et al., 2014; Patzelt et al., 2014; Hagemann et al., 2015; Williams et al., 2016). Regarding heterocystous cyanobacteria, *Nostoc* spp. and *Scytonema* spp. are widely distributed in all types of biocrusts (Büdel et al., 2016, and references therein). Two additional heterocystous genera observed in biocrusts are *Tolypothrix* and *Calothrix* (e.g.,  Garcia-Pichel et al., 2013; Dojani et al., 2014; Williams et al., 2016). The unicellular genus *Chroococcidiopsis* has been reported from biocrusts of different continents, dominating green hypoliths (Büdel et al., 2016). However, few studies have analyzed the cyanobacterial diversity of biocrusts in the Mediterranean (Maestre et al., 2006; Maestre et al., 2011; Muñoz-Martín et al., 2019; Cano-Díaz et al., 2018), and even fewer in the driest areas, despite their important ecological role in these ecosystems and their vulnerability to climate change (Guiot & Cramer, 2016). Therefore, we analyzed key cyanobacterial species in biocrust communities from three dryland areas in southeastern Spain. These sites exhibit key spatial distributions of biocrusts, have contrasted soil texture and lithology and have a gradient of soil stability conditions. They include a completely disturbed system (the Gádor quarry) that is primarily covered by incipient biocrusts (approximately 10% of the surface), and very scarce small plants, a badlands system (El Cautivo) with very active erosion processes in which biocrusts represent more than 50% of the ground cover, and a relatively stable steppe ecosystem (Las Amoladeras) where biocrusts represent almost a third of the soil cover (Chamizo et al., 2012a).

Reliable characterization of cyanobacteria typically requires previous isolation from biocrusts and a morphological analysis by microscopic observation of isolated cultures. The traditional morphological approach is often dependent on identifying specific features that are not always easy to recognize or may vary due to the well-known phenotypic plasticity of cyanobacteria (Komárek, 2016). The development of molecular approaches has led to the use of molecular markers, such as the small ribosomal subunit RNA gene, to complement taxonomic assignations and to infer phylogenetic relationships. In addition, the different cyanobacterial morphotypes and genotypes are ecologically delimited, and their occurrence is determined by the presence of suitable ecological conditions (Komárek, 2018). Thus, the polyphasic approach, which includes assessing morphological, phylogenetic and ecological features, results in a more complete characterization of cyanobacterial strains (Norris & Castenholz, 2006; Komárek, 2016; Jung et al., 2018). Therefore, we carried out a polyphasic approach in which we combined morphological and molecular (16S rRNA gene) information, while also taking into account the environmental conditions of the habitat from which they were isolated. This study addressed the question of whether cyanobacteria inhabiting the driest ecosystems in Europe are similar to those previously reported in biocrusts worldwide.

## Materials and Methods

### Study area

**Figure 1:**
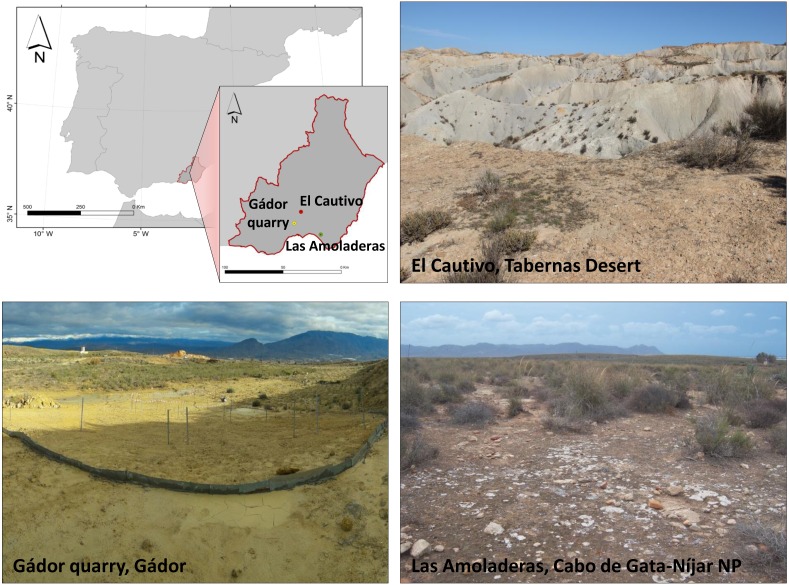
Location and general view of sampling sites. Photo credit: Y Cantón and J R Román.

Samples were collected at three sites located in southeastern Spain: the Gádor quarry, El Cautivo and Las Amoladeras site (Fig. 1).

The Gádor quarry site is a limestone quarry located at the southeastern edge of the Gádor massif (36°55′20″N, 2°30′29″W). The climate is semiarid Mediterranean with a mean annual temperature of 17.6 °C, an annual rainfall of 242 mm and an annual potential evapotranspiration of 1,225 mm (Luna et al., 2016b). Two types of parent rocks are present in the quarried area, calcareous sandstones over Tortonian (upper Miocene) and calcitic-gypsiferous mudstones (Luna et al., 2016a; Román et al., 2018). The soils in the surrounding zones that have not yet been quarried are primarily Leptosols (FAO-IUSS-ISRIC Working Group WRB, 2014). Soil texture of quarried soils is clay loam (34% sand, 31% silt and 35% clay). The soil organic carbon content (SOC) of the substrate on the quarried areas is low even in the top soil with about 1.12 g Kg^−1^, 0.21 g Kg^−1^ of total nitrogen and 0.39 g Kg^−1^ of total phosphate. Average values of pH and electrical conductivity are 8.86 and 1.18 dS m^−1^, respectively (Luna et al., 2016b). Native vegetation colonizing the site before mining consisted primarily of patchy grassland (dominated by *Macrochloa tenacissima* L. Kunth) and some dwarf shrubs (such as *Anthyllis cytisoides*, *Anthyllis terniflora*, *Thymus hyemalis*, *Ulex parviflorus*) or a combination of both plant patches. *Pistacia lentiscus*, *Maytenus senegalensis* and *Rhamnus lycioides* can also be observed (Luna et al., 2016a).

El Cautivo (37°00′38.6″N, 2°26′30.2″W) experimental site is a badlands landscape located in the Tabernas desert that is surrounded by the Alhamilla, Filabres, Nevada and Gádor Mountain Ranges. The climate is semiarid Mediterranean, characterized by a mean annual precipitation of 235 mm, falling primarily in winter, and with long and dry summers. The mean annual temperature is 17.8 °C, and annual potential evapotranspiration is approximately 1,500 mm. The parent material consists of gypsum-calcareous mudstones and calcaric sandstones. The percentage of calcite and gypsum varies from 20 to 35% and 5 to 20%, respectively (Cantón, Solé-Benet & Lázaro, 2003). According to FAO-IUSS-ISRIC Working Group WRB (2014), the soils in this region are primarily Leptosols, Calcisols, Cambisols and Gypsisols, and the soil texture is silty loam (30% sand, 59% silt, and 11% clay) (Chamizo et al., 2015). pH is 7.6 and the electrical conductivity is 1.09 S m^−1^ (Chamizo et al., 2012a). The average SOC of the top soil is 9.4 g Kg^−1^ (Miralles et al., 2012), and total nitrogen content is 0.97 g Kg^−1^. In this area, the active erosion processes have conformed a badlands system (Cantón, Solé-Benet & Lázaro, 2003). The SW-facing slopes are very steep (slope gradients from 30 to 77°) with little soil development, and they are practically devoid of biocrusts or vascular vegetation (Cantón et al., 2004). In contrast, the NE-facing slopes (slope gradients from 10 to 40°) are densely covered by vascular plants and biocrusts in the intershrub spaces. The upper part of these NE-facing slopes show low vegetation cover and intershrub spaces that are primarily covered by lichen dominated-biocrusts. Vegetation cover (primarily *Macrochloa tenacissima* L. Kunth, *Helianthemum almeriense*, *Artemisia barrelieri*, *Salsola genistoides*, and *Euzomodendron bourgaeanum*) increases towards the lower part of the hill slope. In the pediments of these hill slopes, soils are covered by annual and perennial plants with biocrusts, primarily dominated by cyanobacteria, which appear in the interplant spaces. In general, physical crusts cover approximately 30% of the soil surface, while biocrusts occupy more than 50% of the soil surface. Cyanobacteria-dominated biocrusts can also appear as almost the only ground cover of specific landforms.

In the Las Amoladeras experimental site, a dissected caliche, is located at the Cabo de Gata-Níjar Natural Park (36°50′1″N, 2°15′8″W). The climate is also semiarid Mediterranean with an average annual temperature of 19 °C and a mean annual precipitation of 200 mm (Chamizo et al., 2012a). Soils are classified as Leptosols and Calcisols (FAO-IUSS-ISRIC Working Group WRB, 2015), and the soil texture is sandy loam (61% sand, 29% silt and 10% clay) (Chamizo et al., 2015). Physicochemical characteristics of these soils are: pH is 7.9, electrical conductivity is 0.25 S m^−1^, organic carbon content is 12 g Kg^−1^ (Chamizo et al., 2012b), and nitrogen content is 1.35 g Kg^−1^. Approximately one third of the soil surface is covered by scattered shrubs, primarily *Macrochloa tenacissima* L. Kunth, with the 30% of the open areas covered by biocrusts at different successional stages, while the resting surface is covered by calcaric outcrops and stones (Chamizo et al., 2012a). The primary types of biocrusts at this site are cyanobacteria-, lichen- and moss-dominated biocrusts.

### Biocrust sampling

Cyanobacterial biocrust samples were collected under dry conditions using Petri dishes (90 mm diameter, 10 mm deep) in the spring of 2013. At each of the three sites, three samples of two biocrust types (or stages of development) were collected, including incipient light and well-developed dark cyanobacterial-biocrusts, to collect cyanobacterial representatives of both types of biocrusts, as their compositions change with the biocrust developmental state (Chilton, Neilan & Eldridge, 2017). Samples were carefully taken to the laboratory and maintained in darkness under dry conditions and room temperature until their use.

### Cyanobacterial isolation and morphological characterization

Topsoil samples (0.1 g) were ground in a mortar with 1.5 mL of BG11_0_ culture medium (Rippka, Deruelles & Waterbury, 1979) and then centrifuged (3,000 g, 30 s). Supernatants (300 µL) and pellets (50 µL) were separately cultured on agar plates (1.5% agar) containing cycloheximide (0.1 mg/mL^−1^) to avoid fungal contamination with different culture media (BG11_0_, BG11, modified CHU10, modified CHU10 without nitrogen, and Allen and Arnon) (Gómez et al., 2009). The plates were incubated in a growth chamber at 28 °C and 20–50 µmol photons m^−2^ s^−1^ for approximately 4 weeks. Each strain was isolated from colonies, selecting single trichomes using pulled capillary pipettes and forceps under a dissecting microscope (Leica, Leica Microsystems, Wetzler, Germany). Selected trichomes were transferred to multiwell plates with liquid BG11_0_ (for heterocystous cyanobacteria) or BG11 (for nonheterocystous cyanobacteria) and were maintained at 28 °C and 20–50 µmol photons m^−2^ s^−1^. In addition, micromanipulation of the samples under the dissecting microscope with watchmaker’s forceps was performed to manually isolate bundles of filaments directly from the biocrusts as previously described (Garcia-Pichel et al., 2013). These bundles were further separated and cleaned by dragging them over solid agarose medium, observed under a compound microscope to confirm the presence of only one morphotype and were subsequently inoculated in multiwell plates with liquid BG11 medium and cycloheximide (0.1 mg/mL^−1^). Once a high amount of biomass was obtained for each strain, they were morphologically characterized for specific features, including the occurrence of specialized cells, sheaths, trichome characteristics or cell and colony morphology, using an Olympus BH2-RFCA photomicroscope (Olympus, Tokyo, Japan). Morphological characterization was based on Komárek & Anagnostidis (2005) and Komárek (2013). After further incubation, pure cultures were transferred to flasks with liquid culture medium and were grown under the same laboratory conditions.

Cultures were named after the site where the strain was isolated followed by a number (CANT from Gádor quarry, CAU from El Cautivo and AM from Las Amoladeras) and included in the culture collection of the Universidad Autónoma de Madrid (UAM).

### DNA isolation and amplification of the 16S rRNA gene

Total genomic DNA was extracted from isolated cultures with an UltraClean^®^ Microbial DNA Isolation Kit (MO BIO Laboratories, Inc., Carlsbad, CA, USA) using a previously described DNA extraction protocol to break the exopolysaccharides surrounding many cyanobacteria cells (Loza et al., 2013). The protocol involved a three-step process consisting of freezing 0.3 µL aliquots of cyanobacterial suspensions of each culture in liquid nitrogen, breaking them down with an adapted drill and melting them in a 60 °C water bath.

The 16S rRNA gene was PCR amplified using the forward primer pA (5′-AGAGTTTGATCCTGGCTCAG-3′) (Edwards et al., 1989) and the reverse B23SR (5′-CTTCGCCTCTGTGTGCCTAGGT-3′) (Lepère, Wilmotte & Meyer, 2000), which produced amplicons of approximately 2,000 bp, under conditions previously described by Mateo et al. (2011) and following the PCR conditions of Gkelis et al. (2005). Amplicons were run in an agarose gel (1%) with a 1 Kb Gene Ruler (MBL Biotools, Spain) and visualized with fluorescent DNA stain Gel Red™ to assess if the size of the amplified product was correct. PCR products were purified using a Real Clean Spin Kit (REAL, Durviz S, L., Valencia, Spain) and were cloned into the pGEM^®^-T Easy Vector (Promega, Madison, USA). The inserts of positive clones were verified by PCR using the primers T7 (5′-TAATACGACTCACTATAGGG-3′) and SP6 (5′-ATTTAGGTGACACTATAG-3′). Plasmids from clones with a confirmed insert (1 or 2 for each strain) were extracted using a Wizard Miniprep kit (Promega, Madison, USA) and commercially sequenced (Genomics Core Unit of the Spanish National Cancer Research Center, Spain) using the aforementioned T7 and SP6 primers and the primer 16S684F (5′-GTGTAGCGGTGAAATGCGTAGA-3′), which was designed in a previous study (Mateo et al., 2011). Partial sequences were aligned into contigs and were manually corrected to remove ambiguous sites using BIOEDIT (version 7.2.5; Hall, 1999). Nucleotide sequences were deposited in the GenBank database under the accession numbers MG641898 to MG641936.

### Phylogenetic analyses

For the phylogenetic analyses, our 16S rRNA gene sequences (1,478–1,490 bp) were compared with sequence information available in the National Center for Biotechnology Information (NCBI) database using BLAST (http://www.ncbi.nlm.nih.gov/BLAST). Assignations with an identity value higher than 97.5% and other representative soil cyanobacteria sequences were subsequently downloaded, and multiple alignments of all these sequences were generated using the Clustal W Multiple Alignment function in BIOEDIT, with the *Escherichia coli* 16S rRNA gene sequence used as an outgroup. The phylogenetic trees were generated using MEGA (version 7.0.21; Kumar et al., 2008). The alignment was checked for the best fitting evolutionary model in MEGA, which determined that the general time reversible (GTR) model with a gamma distribution of rate variation was the most appropriate model. Therefore, distances for the maximum likelihood (ML) tree were estimated by the GTR Model, assuming a gamma distribution with four categories with the Nearest-Neighbor-Interchange. For neighbor joining, evolutionary distances were calculated using the Tajima-Nei model (Tajima & Nei, 1984) with a pairwise deletion of gaps and missing data. The standard error was estimated with the bootstrap phylogeny test (Felsenstein, 1985) using 1,000 replications, which was also used for the ML tree. The Maximum Parsimony tree was generated using the Subtree-Pruning-Regrafting search method, with ten initial trees and three search level. Gaps and missing data were treated with the complete deletion option and the standard error was calculated via the bootstrap method using 100 replications. The percent similarity between sequences was determined as (1-p-distance)*100.

## Results

**Figure 2:**
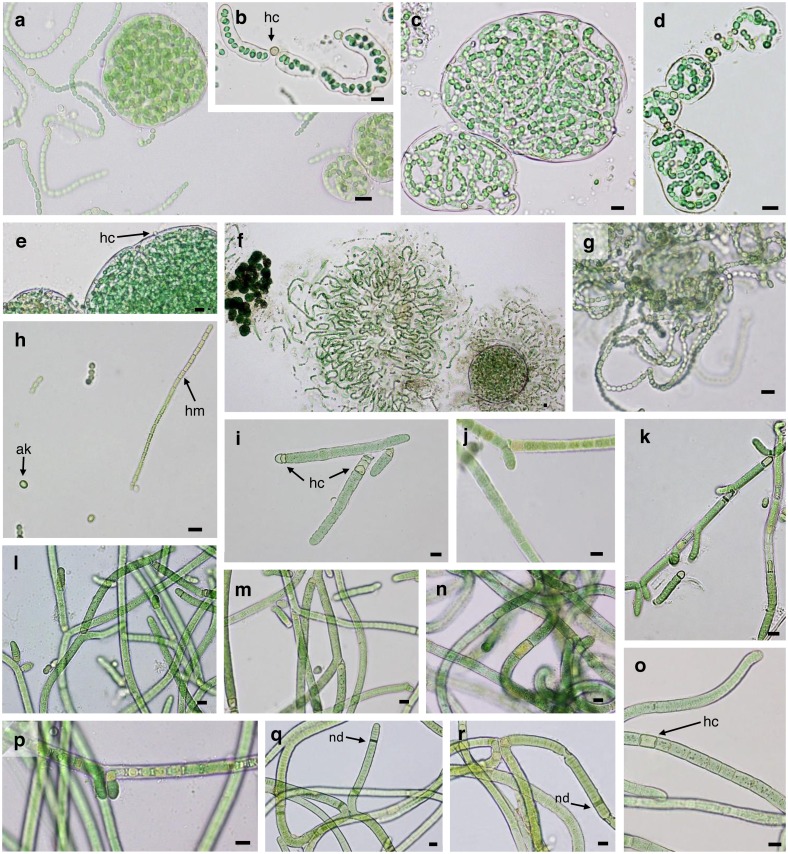
Microphotographs of heterocystous cyanobacteria. (A–C) *N. commune* CANT2. (D–F) *N.  commune* CANT4. (G and H) *N. calcicola* AM50. (I) *T. distorta* CANT1.(J) *T. distorta* CANT6. (K) *T. distorta* CANT7. (L) *T. distorta* CAU3. (M) *T. distorta* CAU12. (N) *S. hyalinum* CAU4. (O and P) *S. hyalinum* CAU6. (Q and R) *S. hyalinum* AM54. Site codes are AM (Las Amoladeras), CAU (El Cautivo) and CANT (Gádor quarry). Scale Bar = 10 µm , hc, heterocyst, ak, akinete, hm, hormogonium, nd, necridia.

**Figure 3:**
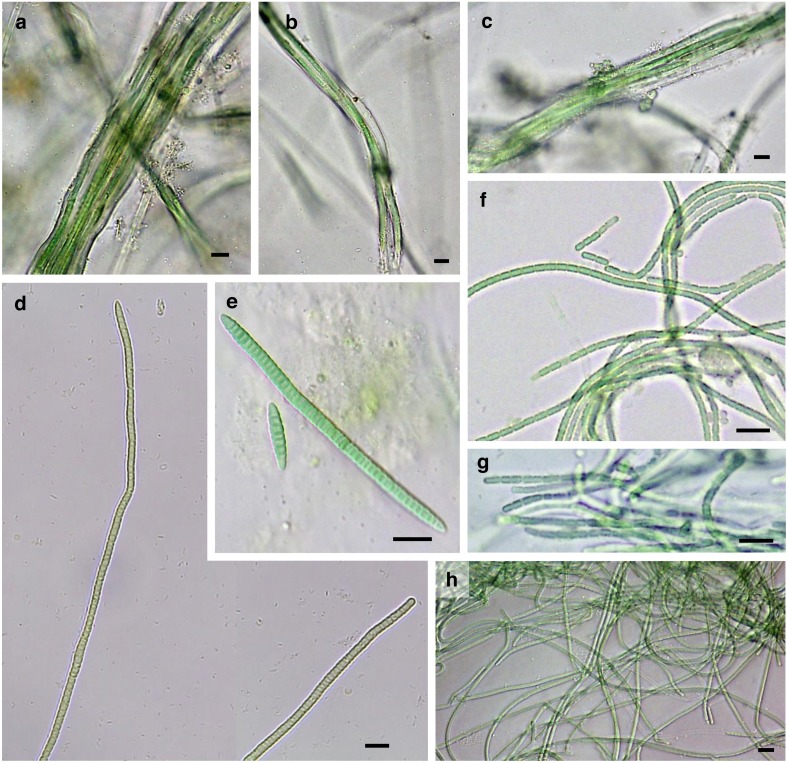
Microphotographs of bundle-forming filamentous cyanobacteria. (A–C) *M. steenstrupii* CAU8. (D and E) *T. desertorum* CAU7. (F) *S.* cf. *calcicola* AM125. (G) *S.* cf. *calcicola* AM57. (H) *S.* cf. c *alcicola* AM116. Site codes are AM (Las Amoladeras), CAU (El Cautivo) and CANT (Gádor quarry). Scale Bar = 10 µm.

**Figure 4:**
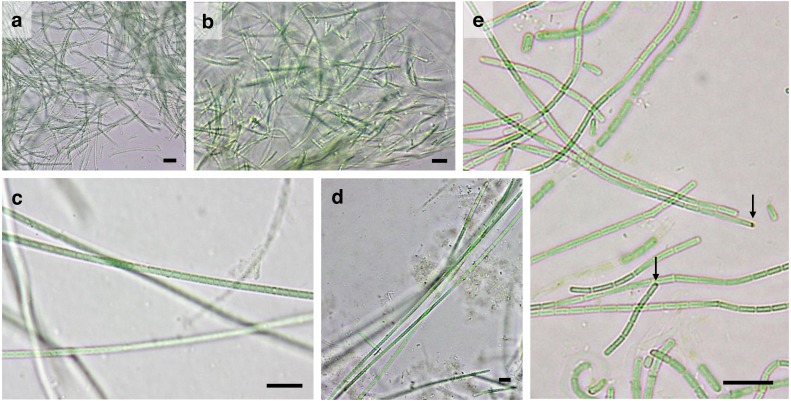
Microphotographs of other filamentous non-heterocystous cyanobacteria. (A) *L. frigida* CANT10. (B) *L. frigida* CANT11. (C and D) *L. frigida* CAU10. (E) *O. kazantipica* AM118. Arrows indicate the characteristic reddish eyespot (oculus) at the tip of mature apical cells in *Oculatella* genus. Site codes are AM (Las Amoladeras), CAU (El Cautivo) and CANT (Gádor quarry). Scale Bar = 10 µm.

Twenty-five strains were successfully isolated, cultured and characterized, including eight from Gádor quarry, eleven from El Cautivo and six from Las Amoladeras (Table 1). The morphological characteristics of these strains are shown in Figs. 2–4 and in Table 2.

**Table 1:** Cyanobacterial strains analyzed in this study.

**Taxon**	**Strain**	**Culture collection no**^a^	**Sampling location**
*Nostoc commune*	CANT4	UAM 816	Gádor quarry
	CANT2	UAM 817	Gádor quarry
*Nostoc calcicola*	AM50	UAM 818	Las Amoladeras
*Scytonema hyalinum*	CAU4	UAM 819	El Cautivo
	CAU6	UAM 820	El Cautivo
	AM54	UAM 821	Las Amoladeras
*Tolypothrix distorta*	CANT1	UAM 822	Gádor quarry
	CANT3	UAM 823	Gádor quarry
	CANT6	UAM 824	Gádor quarry
	CANT7	UAM 825	Gádor quarry
	CAU1	UAM 826	El Cautivo
	CAU3	UAM 827	El Cautivo
	CAU12	UAM 828	El Cautivo
	CAU13	UAM 829	El Cautivo
	CAU14	UAM 830	El Cautivo
*Microcoleus steenstrupii*	CAU8	UAM 831	El Cautivo
*Trichocoleus desertorum*	CAU7	UAM 832	El Cautivo
*Schizothrix* cf. *calcicola*	AM57	UAM 833	Las Amoladeras
	AM116	UAM 834	Las Amoladeras
	AM125	UAM 835	Las Amoladeras
*Oculatella kazantipica*	AM118	UAM 836	Las Amoladeras
*Leptolyngbya frigida*	CAU10	UAM 837	El Cautivo
	CAU11	UAM 838	El Cautivo
	CANT10	UAM 839	Gádor quarry
	CANT11	UAM 840	Gádor quarry

**Notes.**

a UAM Culture Collection, Universidad Autónoma de Madrid, Spain.

**Table 2:** Morphological characteristics of cells in cyanobacterial taxa observed in this study. Measurements are given as mean ± standard deviation/range for *n* = 100.

**Taxon**	**Breath (µm)**	**Length (µm)**	**Figure**
*Nostoc commune*	**VC**: 3.4 ± 0.5/2–5	**VC**: 3.7 ± 0.6/2.1–5.4	Figs. 2A–2F
	**H**: 4.4 ± 0.5/3–5.3	**H**: 5 ± 0.7/3.5–6.3	
*Nostoc calcicola*	**VC**: 3.4 ± 0.3/2.5–4	**VC**: 4.1 ± 0.6/3.–5.4	Figs. 2G–2H
	**H**: 4.2 ± 0.6/3.1–5.3	**H**: 4.7 ± 0.8/3.4–6.3	
*Scytonema hyalinum*	**VC**: 8.5 ± 1.4/5.2–12	**VC**: 8.7 ± 3.6/3.7–20.1	Figs. 2N–2R
	**H**: 9 ± 1.9/4.8–12.3	**H**: 12.2 ± 4.5/6.9–23.1	
*Tolypothrix distorta*	**VC**: 8.8 ± 1.3/5.6–12.1	**VC:** 6.8 ± 1.9/3.2–12.8	Figs. 2I–2M
	**TH**: 7.6 ± 1.2/5.8–9.2	**TH**: 5.6 ± 1/3.3–7.3	
	**IH**: 7.1 ± 1.5/4.6–9.7	**IH:** 9.6 ± 2.3/5.1–15.1	
*Microcoleus steenstrupii*	**VC**: 3.8 ± 0.4/3.3–4.4	**VC**: 3.8 ± 0.8/2.1–6	Figs. 3A–3C
*Trichocoleus desertorum*	**VC**: 2.8 ± 0.4/1.8–3.8	**VC**: 2 ± 0.5/0.9–3.6	Figs. 3D–3E
*Schizothrix* cf. calcicola	**VC**: 1.8 ± 0.2/1.2–2.3	**VC**: 2.9 ± 0.7/1.6–4.8	Figs. 3F–3H
*Oculatella kazantipica*	**VC**: 1.2 ± 0.1/0.8–1.6	**VC**: 2.7 ± 0.6/1.5–4.1	Fig. 4E
*Leptolyngbya frigida*	**VC**: 1.4 ± 0.2/1.1–1.8	**VC**: 2.1 ± 0.4/1–3.5	Figs. 4A–3D

**Notes.**

VC vegetative cells H heterocysts H heterocysts TH terminal heterocysts IH intercalary heterocysts

**Figure 5:**
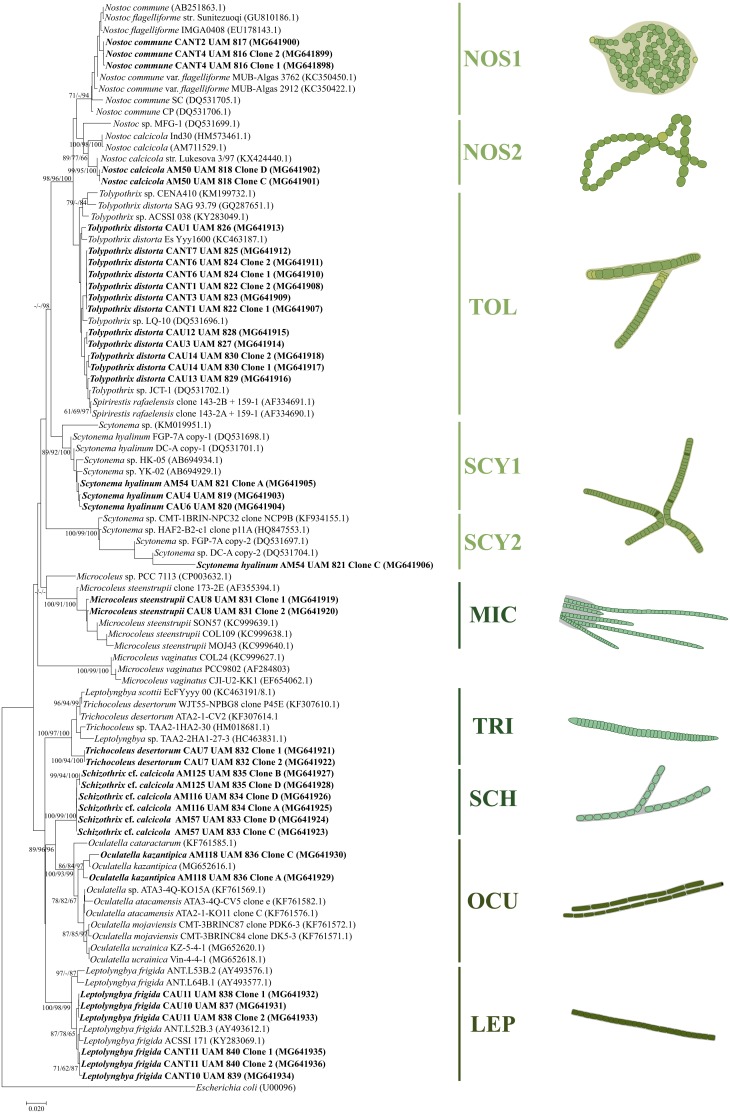
Phylogenetic tree based on 16S rRNA gene sequences obtained by the Neighbor Joining method. The percentage of trees in which the associated taxa clustered together (Bootstrap) is shown next to the branches (>60% values are reported for Neighbor Joining, Maximum Likelihood and Maximum Parsimony analysis). The tree is drawn to scale, with branch lengths measured in the number of substitutions per site. The 0.02 bar indicates substitutions per nucleotidic position. Newly sequenced strains are in bold. Different colors correspond to different types of observed cyanobacteria. Yellowish-green: heterocystous cyanobacteria; blue-green: bundle-forming filamentous cyanobacteria; green: other filamentous nonheterocystous cyanobacteria.

The phylogenetic analysis shows well-defined clusters corresponding to the 9 cyanobacterial species identified (Fig. 5). The phylogenetic tree was constructed from 39 16S rRNA gene sequences from our isolated cyanobacterial cultures and 54 16S rRNA gene sequences from the NCBI database corresponding to soil cyanobacterial strains. All the algorithms used to construct the phylogenetic tree distributed the sequences in 10 different clusters according to the observed morphotypes described below (Fig. 5).

### Heterocystous cyanobacteria

Three of the strains isolated from the biocrust samples showed characteristics typical of the *Nostoc* genus (Figs. 2A–2H). Two Gádor quarry isolates (CANT2 and CANT4) exhibited a 99.7% sequence similarity and were included in the phylogenetic tree along with *Nostoc commune* sequences from the NCBI database in cluster NOS1 (Fig. 5), which exhibited over a 99% sequence similarity to a representative sequence of this taxon. The observed phenotypic features and occurrence of these strains also fit this morphotype (see Komárek, 2013 and discussion below). The isolates presented almost spherical (2.5–5 µm wide, 2–5.4 µm long) or barrel-shaped (2–3.7 µm wide, 2.7–5.3 µm long) and very constricted cells, forming either densely entangled trichomes that were observed either individually or together and surrounded by a clearly visible sheath (Figs. 2A–2F). Nearly spherical heterocysts (3–5.3 µm wide, 3.5–6.3 µm long) (see Figs. 2B and 2E) appeared in either a terminal or intercalary position. Therefore, these strains were identified as *N. commune*.

Strain AM50 was isolated from a biocrust sampled at Las Amoladeras. The morphology of this strain differed from the *Nostoc* strains isolated from the Gádor quarry samples (Figs. 2G–2H). Cultures showed freely entangled filaments with no distinguishable sheaths that contained barrel-shaped cells (2.5–4 µm wide and 3–5.4 µm long), spherical heterocysts (3.1–5.3 µm diameter) and slightly elongated akinetes (4–5 µm wide and 6–7 µm long) (Fig. 2H, Table 2). Hormogonia with more compressed, 2.5–3.8 µm wide and 3–4 µm long quadrate cells were also observed (Fig. 2H). The 16S rRNA gene sequences that were 99.72% similar to *Nostoc calcicola* from the NCBI database, were included in cluster NOS2 (Fig. 5). Thus, the morphological characteristics fitting those of *N. calcicola* (Komárek, 2013), the habitat it was isolated from, and phylogenetic analysis supported this strain as belonging to this species.

Nine strains isolated from biocrusts sampled at El Cautivo (CAU1, CAU3, CAU12, CAU13 and CAU14) and the Gádor quarry (CANT1, CANT3, CANT6 and CANT7) were initially classified as belonging to *Tolypothrix* because their morphological characteristics, which were typical of this genus, with falsely branched filaments observed that were typically single branches growing mostly in the direction of the original filament and often originating below intercalary heterocysts (Figs. 2I–2M, Table 2). Cells were typically shorter than they were wide or cylindrical (5.6–12.1 µm wide, 3.2–12.7 µm long) with colorless sheaths (Figs. 2I–2M). Trichomes were slightly constricted, with many false branches diverging from the primary filament at approximately 45° and had rounded terminal cells. Heterocysts were wider than they were long (7.7–11 µm wide and 3.3–7.7 µm long) (Figs. 2I–2M), solitary, although sometimes in pairs, and were typically observed at the bases of branches (Fig. 2J). The 16S rRNA gene sequences of all strains (all strains similarities were 98.4–100%) were included in a cluster (TOL, Fig. 5) with other *Tolypothrix* sp., *Tolypothrix distorta* and *Spirirestis rafaelensis* sequences, and they were 98.3–99.1% similar to *T. distorta* from the NCBI database. *S. rafaelensis* is a soil cyanobacterial species with morphological resemblance to *T. distorta* but with spiral-shaped filaments (Flechtner et al., 2002). Since our strains did not exhibit this feature and matched the description of *T. distorta*, and taking into account the ecological characteristics of the sites where they were observed, they were assigned to this taxon.

Strains belonging to the genus *Scytonema* were identified in biocrusts from El Cautivo (CAU4 and CAU6) and Las Amoladeras (AM54). All these strains had similar morphological features characteristic of this genus: (i) Common double false branches with the same width as the primary filaments originating between vegetative cells and typically slightly distant from the heterocysts (Figs. 2N–2R); (ii) nonconstricted cylindrical cells (5.2–12 µm wide × 3.7–20.1 µm long) forming filaments with colorless sheaths and rounded terminal cells; and (iii) heterocysts, also cylindrical (4.8–12.3 µm wide and 6.6–23.1 µm long), and typically solitary (see Figs. 2N and 2O). Numerous necrids were also observed (e.g., Figs. 2Q–2R). Sequencing after cloning yielded two divergent 16S rRNA gene copies for strain AM54 (Copies 1 and 2, Fig. 5), located in separate clades together with previously characterized divergent copies of *Scytonema hyalinum* from North American biocrusts, corresponding to divergent intragenomic operons (Yeager et al., 2007; Johansen et al., 2017). The isolated strains CAU4 and CAU6 yielded only one sequence, which was 99.7% similar to AM54 Copy 1. Therefore, these strains were identified as belonging to *S. hyalinum.*

### Bundle-forming filamentous cyanobacteria

Several strains were included in the genera *Microcoleus*, *Trichocoleus* and *Schizothrix*, which are known to be able to form bundles. Two were isolated from biocrusts sampled at El Cautivo (CAU7 and CAU8) and the rest were isolated at Las Amoladeras (AM116, AM57 and AM125).

For the isolated strain CAU8, typical bundles formed by trichomes in groups surrounded by a common colorless sheath could be observed (Figs. 3A–3C) that fit the morphotype of *Microcoleus steenstrupii* sensu Boyer et al. (2002). Cells were cylindrical (3.3–4.4 µm wide) with classical phormidiacean cell division, when discernible, in which cells grow to full size before new cell walls begin to form. Terminal cells were conical without calyptra. The 16S rRNA gene sequences were clustered together with sequences of *M. steenstrupii* from North American desert biocrusts (cluster MIC, Fig. 5) and exhibited 97.4–99.2% similarities within this cluster; thus, this strain was identified as belonging to that taxon.

The isolated strain CAU7 showed morphological characteristics of the recently described species *Trichocoleus desertorum*, which was isolated from biocrusts of arid deserts (Mühlsteinová et al., 2014). In addition, the 16S rRNA gene sequences were also included in the phylogenetic tree together with sequences corresponding to this species (Cluster TRI, Fig. 5), exhibiting a 97.7% sequence similarity. Therefore, this strain was identified as belonging to this taxon. Cells were typically wider than they were long (1.8–3.8 µm wide) and formed trichomes alone or in bundles surrounded by a colorless sheath. Terminal cells were rounded or conical without calyptra, and cells were slightly constricted, with some harboring inclusions (Figs. 3D–3E).

General morphological features that fit the taxonomic descriptions of *Schizothrix calcicola* (see Komárek & Anagnostidis, 2005) could be observed in the isolated strains AM57, AM125 and AM116 (Figs. 3F–3H). Filaments were typically entangled, curved and sometimes had false branches and nonconical terminal cells. Sheaths were colorless and often contained only a single trichome, sometimes two. Cells were longer than they were wide (1.2–2.3 µm wide and 1.6–4.8 µm long) and slightly constricted, with cross-walls that were not narrowed (Figs. 3F–3H). The 16S rRNA gene sequences were included in cluster SCH (Fig. 5), with sequence similarities ranging from 99.7 to 100%, although no matches were identified by the BLAST search of the NCBI database. *S. calcicola* is a terrestrial species that is commonly observed in biocrusts (see ‘Discussion’ below), and therefore are ecologically similar to our isolates. In addition, they were isolated directly by micromanipulation of bundles from biocrust samples. Therefore, these isolates were therefore characterized as *Schizothrix* cf. *calcicola*.

### Other filamentous nonheterocystous cyanobacteria

Narrow filamentous cyanobacteria belonging to the genera *Leptolyngbya* or *Oculatella* were present at all sites, four of which were isolated from El Cautivo (CAU 10 and CAU11) and the Gádor quarry (CANT10 and CANT11). Phylogenetic analyses placed all of these 16S rRNA sequences in cluster LEP (Fig. 5) together with *Leptolyngbya frigida* sequences (formerly *Pseudanabaena frigida*) from the NCBI database, with similarities of 97.7–99.5% observed within the cluster. As the morphology of these isolates also fit with those of this taxon (Komárek & Anagnostidis, 2005), and because it was identified in ecologically similar localities (see discussion below), they were identified as belonging to this species. The cells of these isolates were cylindrical (1–1.8 µm wide, 1-3.5 µm long) and constricted with translucent cross-walls (Figs. 4A–4D). Aerotopes were occasionally observed near the cell extremes. Filaments were entangled, curved, with rounded apical cells and colorless sheaths (Figs. 4A–4D). The strain isolated from Las Amoladeras biocrusts (AM118) yielded a 16S rRNA gene sequence that was included in cluster OCU, which also included *Oculatella* sequences (Fig. 5). The genus *Oculatella*, which is morphologically similar to the genus *Leptolyngbya*, was separated from the latter based on genetic differences (Zammit, Billi & Albertano, 2012; Osorio-Santos et al., 2014). In addition, this genus has a characteristic reddish eyespot (oculus) at the tip of mature apical cells for which the genus was named, and this feature could be observed in our cultures (Fig. 4E). Recently, new species of *Oculatella* were reported from terrestrial habitats of Ukraine (Vinogradova et al., 2017). Our isolate has similar morphological characteristics to one of these species, *Oculatella kazantipica*, exhibiting cylindrical cells that are longer than they are wide (0.8–1.6 µm wide and 1.5–4.1 µm long), are slightly constricted and with invisible cross-walls, and are rarely associated with granules (Fig. 4E, Table 2). Trichomes were moderately entangled, with rounded apical cells enclosed by a colorless sheath. In addition, the 16S rRNA analysis showed a 98.9% similarity of this strain with the new aforementioned species from the Ukrainian biocrust. Therefore, our isolate was assigned to this taxon.

The majority of identified species were found in more than one site. However, Las Amoladeras completely differed in the isolated species, whereby *N. calcicola, S.* cf. *calcicola,* and *O. kazantipica* were only found at this location.

## Discussion

Cyanobacteria are the main primary producers and the major photosynthetic soil colonizers in biocrusts (Garcia-Pichel & Wojciechowski, 2009). The identification of relevant cyanobacteria in typical biocrusts of arid and semiarid zones has significant implications, since an understanding of the differences in microbial composition in biocrusts from different regions will be crucial for managing these communities (Belnap, 2013). In addition, there is a need to identify which microorganisms are present in Mediterranean ecosystems, as they have been identified as one of the most prominent hotspots in future climate change projections (Guiot & Cramer, 2016). In this study, we used a polyphasic approach in which we combined morphological, molecular, and ecological habitat data, which enabled us to identify key soil cyanobacterial species inhabiting biocrusts from three different sites in southeastern Spain where biocrusts are significant ecosystem components. Although a large number of studies on cyanobacterial diversity in biocrusts have been performed worldwide, especially in North America (Büdel et al., 2016), this is, to the best of our knowledge, the first study to compare molecular and morphological characteristics of cyanobacteria from representative biocrusts of the driest European Mediterranean region. Interestingly, most of the cyanobacterial strains identified in this study were different from those identified in another recent study at a gypsiferous site in central Spain (Aranjuez), which is also in the Mediterranean region but is more humid (Cano-Díaz et al., 2018). This difference agrees with previous studies comparing distinct soil types, which have revealed that cyanobacterial communities in gypsum soils are different from those at calcareous sites (Garcia-Pichel, Lopez-Cortes & Nubel, 2001; Steven et al., 2013). This observation explains the observed differences in Las Amoladeras biocrusts, as the lithology at this site is calcareous. El Cautivo and Gádor quarry sites, which were the most similar to each other, despite being a gypsiferous mudstone, are very different from Las Amoladeras and Aranjuez. At both, El Cautivo and Gádor quarry sites, the parent material has an approximate 20% gypsum content. However, in the upper soil horizons it is lower (from 0.5 to 5%) due to leaching and runoff, which is why cyanobacterial species colonizing these sites must be able to tolerate gypsum.

Comparing the cyanobacterial crust composition from other regions in Europe, clear differences were observed. Williams et al. (2016) described the cyanobacterial diversity of biocrusts across a latitudinal gradient of Western Europe, observing that of the 19 morphologically identified genera, *Nostoc*, *Oscillatoria*, *Pseudanabaena*, *Phormidium* and *Microcoleus* were present at all the sites. Furthermore, the use of the next-generation sequencing culture-independent approach demonstrated that site variation was substantial, but all were dominated by *Leptolyngbya*, *Phormidium* and a cyanobacterial taxon that could not be further identified from the utilized databases (Williams et al., 2016). However, comparisons with other geographical locations worldwide, specifically with some sites in the aforementioned European study, showed similarities in species composition (see below).

### Heterocystous cyanobacteria

*N. commune* is a cosmopolitan, widely distributed cyanobacterium in desert biocrusts (Evans & Johansen, 1999) that has been genetically identified in North America (Garcia-Pichel, Lopez-Cortes & Nubel, 2001; Yeager et al., 2007), southwestern Africa (Büdel et al., 2009), Chinese deserts (Zhang et al., 2016), and in semiarid soils from Australia and Spain (Williams et al., 2014; Aboal et al., 2016). This species has also been morphologically identified in biocrusts from North American (Flechtner, Johansen & Clark, 1998; Belnap & Lange, 2003) and Chinese deserts (Scherer & Zhong, 1991). *N. calcicola* has been morphologically and genetically identified in biocrusts from South Africa and Namibia by Dojani et al. (2014) and by Büdel et al. (2009). The *Nostoc* genus was also observed to be dominant at El Cautivo site in previous studies (Williams et al., 2016).

*T. distorta* is commonly observed in biocrusts worldwide, although in some areas a similar taxon, *S. rafaelensis*, has been described that differs from *T. distorta* only in that the filaments are regularly, tightly coiled in a right-handed helix (Flechtner et al., 2002), but have a 16S rDNA sequence that is over 99% similar to *T. distorta*, making these taxa genetically indistinguishable. However, the morphological features of our strains enabled us to discard the identification of this taxon as *S. rafaelensis*, since no spiral morphotype was observed. The *T. distorta* phylotype has been previously observed in biocrusts from North America (Flechtner et al., 2002; Yeager et al., 2007), South Africa and Namibia (Büdel et al., 2009; Dojani et al., 2014) and the *T. distorta* morphotype has also been described in North America (Flechtner, Johansen & Belnap, 2008).

*S. hyalinum* has previously been genetically identified in biocrusts from arid sites in South Africa and Namibia (Dojani et al., 2014), North America (Yeager et al., 2007; Yeager et al., 2012) and the hyperarid Atacama Desert (Patzelt et al., 2014), and morphologically identified in North America (Flechtner et al., 2002; Flechtner, Johansen & Belnap, 2008). *Scytonema* sp. has also been observed in China (Zhang et al., 2016), in the Negev Desert (Hagemann et al., 2015), recently in Chile (Baumann et al., 2018), and in Europe, where it has only been reported at the Tabernas Desert (Williams et al., 2016).

### Bundle-forming cyanobacteria

Of the bundle-forming cyanobacteria, *M. steenstrupii* has also been genetically and morphologically characterized in North and Central America (Flechtner, Johansen & Clark, 1998; Boyer et al., 2002; Nagy, Pérez & Garcia-Pichel, 2005; Garcia-Pichel et al., 2013) and China (Zhang et al., 2016). *T. desertorum* has been morphologically and genetically identified in biocrusts from the Atacama, Mojave and Colorado Deserts (Mühlsteinová et al., 2014) and the Gurbantunggut Desert (Zhang et al., 2016). The morphology of the strains isolated from biocrusts sampled at the Las Amoladeras site fit the description of *S. calcicola*, but the 16S rDNA sequences were placed alone in a cluster (SCH) with no matches in the databases. Thus, we identified a novel biocrust-associated phylotype of *Schizothrix*. *S. calcicola* has also been morphologically identified in North American desert biocrusts (Flechtner, Johansen & Clark, 1998; Belnap & Lange, 2003). In addition, according to Williams et al. (2016), the genus *Schizothrix* was not observed in a biocrust sampling study across a European gradient, including the Tabernas Desert, except for the Nature Reserve Gynge Alvar in Sweden. Thus, *Schizothrix* spp. must be adapted to very specific environments, as it is not a common genus within European biocrusts.

### Other filamentous nonheterocystous cyanobacteria

Other filamentous nonheterocystous cyanobacteria commonly observed in biocrusts were represented by our *L. frigida* and *O. kazantipica* isolates. *L. frigida*, originally thought to be endemic to Antarctic freshwaters (Komárek et al., 2015), has been observed in South African (Büdel et al., 2009; Dojani et al., 2014), Arctic and European biocrusts (Jancusova et al., 2016). In addition, the genus *Oculatella* had only reported from Mediterranean countries (Zammit, Billi & Albertano, 2012), but further studies showed a wide distribution, as it has been observed in North and South America (Osorio-Santos et al., 2014), from biocrusts in Iran (Dulić et al., 2017) and Ukraine, where *O. kazantipica* was described as a new species (Vinogradova et al., 2017), and recently from biocrusts of the Arctic (Jung et al., 2018).

### Implications for ecosystem services

The cyanobacteria identified in this study represent keystone pioneer organisms in the Mediterranean Basin, a hotspot of biodiversity, where they perform crucial ecological services that support ecosystem health.

Heterocystous cyanobacteria, such as those of the genera *Nostoc*, *Tolypothrix* and *Scytonema* observed in this study, have essential ecological functions in drylands. Dinitrogen fixation is an important process in soil biocrusts that has been estimated to be responsible for nearly 30% of the total nitrogen fixed in terrestrial ecosystems (Yeager et al., 2012), primarily by heterocystous cyanobacteria (Barger et al., 2016). Furthermore, cyanobacteria notably increase soil fertility through carbon sequestration (Yan-Gui et al., 2013). Therefore, in low-nutrient environments with few symbiotic nitrogen-fixing vascular plants, biocrust-associated cyanobacteria have important roles in ecosystem N and C cycling (Barger et al., 2016; Büdel et al., 2016; Rodríguez-Caballero et al., 2018).

The bundle-forming cyanobacteria *M. steenstrupii*, *T. desertorum* and *S.* cf. *calcicola* have been described as pioneering biocrust colonizers that stabilize soils through the cellular web they form and the secretion of polysaccharides, making these microhabitats erosion resistant (Garcia-Pichel & Wojciechowski, 2009) and allowing the subsequent colonization of heterocystous cyanobacteria, and later colonization by lichens and mosses (Weber et al., 2016).

It is worth mentioning that *M. vaginatus*, one of the most widespread soil cyanobacteria in the world, was not identified in this study. Although we cannot rule out unsuccessful isolation, the absence of these cyanobacteria among the isolates obtained in this study may be related to the warm and dry environmental conditions of the study sites. *M. vaginatus* dominates cool desert soils, whereas *M. steenstrupii* is a thermotolerant species that has been described as dominant in biocrusts in hot deserts (Gundlapally & Garcia-Pichel, 2006; Garcia-Pichel et al., 2013), replacing the dominance by the former species (Garcia-Pichel et al., 2013). In addition, similar ecological functions may be performed by *S.* cf. *calcicola*, which also forms bundles that contribute to soil stabilization, with this species observed to be more abundant in biocrusts in warm and dry locations than in low-temperature sites that correspond to its thermotolerant physiology (Muñoz-Martín et al., 2019). *S. hyalinum* has also been reported as being thermotolerant, a species that is capable of increasing its abundance in the warmest and driest locations in Spain (Muñoz-Martín et al., 2019) and developing specialized cells, such as akinetes (spore-like), that could also lead to a higher tolerance to warmer and drier locations (Hu, Gao & Whitton, 2012), such as the environmental conditions observed in this study.

### Concluding remarks

In this study, key soil cyanobacteria inhabiting biocrusts from three representative sites of the most arid region in Europe (southeastern Spain) were characterized through a polyphasic approach, which has currently been highlighted by various authors as the most reliable option for identifying cyanobacteria. The results of this study increase our understanding of which cyanobacterial species colonize biocrusts in the most arid region in Europe. Understanding differences in microbial composition in soils from different geographical regions will enable better management and restoration of dryland ecosystems. In addition, incorporating the new sequences obtained in this study into public databases, together with the morphological characteristics and ecological habitat data of the associated strains, provides valuable information for future research, allowing further comparisons in culture-independent analyses.

##  Supplemental Information

10.7717/peerj.6169/supp-1 Supplemental Information 1 DNA Sequencing

## Additional Information and Declarations

### Data Availability

The following information was supplied regarding data availability: Nucleotide sequences are available in the GenBank database under the accession numbers: MG641898 to MG641936.
